# Inflammatory memory restrains intestinal stem cell regeneration

**DOI:** 10.21203/rs.3.rs-2566520/v1

**Published:** 2023-03-07

**Authors:** Pavan Reddy, Dongchang Zhao, Visweswaran Ravikumar, Emma Lauder, Lu Li, Yaping Sun, Katherine Oravecz-Wilson, Michael Brooks, Evan Keller, Fengju Chen, Laure Maneix, Ana Santibanez, Chad Creighton, Arvind Rao

**Affiliations:** Baylor College of Medicine; Baylor College of Medicine; University of Michigan; Baylor College of Medicine; Baylor College of Medicine; Baylor College of Medicine; U of Michigan; Washington University School of Medicine; University of Michigan; Baylor College of Medicine; Baylor College of Medicine; Baylor College of Medicine; Baylor College of Medicine; University of Michigan

## Abstract

Intestinal stem cells (ISC) encounter inflammatory insults in immune mediated gastro-intestinal (GI) diseases. It remains unknown whether, and how, they adapt, and if the adaptation leaves scars on the ISCs that affects their subsequent regeneration capacity. We investigated the consequences of inflammation on Lgr5^+^ISCs in well-defined clinically relevant models of gastro-intestinal acute graft-versus-host disease (GI GVHD). Utilizing single cell transcriptomics, organoid, metabolic, epigenomic and *in vivo* models we found that Lgr5^+^ISCs undergo metabolic changes that lead to accumulation of succinate, which reprograms its epigenome. These changes reduced the ability of ISCs to differentiate and regenerate *ex vivo* in serial organoid cultures demonstrating the persistence of the maladaptive impact of an *in vivo* inflammatory encounter by the ISCs. Thus, inflammation from GI GVHD leaves a memory of its effects on ISCs that persist and are likely to affect their sensitivity to adapt to future stress or challenges.

## One-sentence Summary

Past exposure to inflammation leaves its imprint on intestinal stem cells and regulates its subsequent functions.

## Introduction

The memory paradigm of non-immune cells, particularly in adult tissue specific stem cells that remember their inflammatory encounters and its impact on their fitness and functions has only been recently appreciated (1–4). However, the molecular mechanisms underpinning this phenomenon, and whether such memories are germane to intestinal stem cells (ISCs) remain unknown. ISCs renew the different cell types of the intestinal epithelial barrier (5). ISCs undergo a multitude of choregraphed changes, demonstrate plasticity and integrate environmental cues to maintain normal intestinal homeostasis (6, 7). Non-infectious causes of intestinal inflammation and injury, specifically gastrointestinal graft-versus-host disease (GI GVHD) target ISCs resulting in their loss and thus cause severe morbidity and mortality from allogeneic hematopoietic stem cell transplantation (Allo-HSCT) (8, 9). GI GVHD is the major cause of mortality and morbidity from Allo-HSCT, a potentially curative therapy for many hematological diseases. The biology of GVHD is complex. GVHD is caused by inflammation that is induced by cytopathic alloreactive T cells that target host epithelial cells in GI tract, skin and liver. This inflammation is amplified or mollified by complex microenvironment characterized by several other host cells and its microbiome (8, 10, 11). The host ISCs are the chief targets of the inflammation that causes GI GVHD (12–15). GI GVHD is characterized by reduction in ISCs. However, it remains unknown whether the ISCs that survive or tolerate inflammation from GVHD are fully functional, or can return to their full functionality after the resolution of GVHD, with fundamental implications for host resilience and repair (16).

Cellular metabolism plays a central role in regulation of ISC function and regeneration (17–23). Emerging data have demonstrated a role for inflammation in causing metabolic adaptations in various immune and non-immune cell targets (24–32). However, whether ISCs show altered metabolism in the context of inflammation induced stress remains unclear. Metabolism has been shown to regulate epigenetic reprogramming (33, 34). Specifically, metabolites derived from oxidative phosphorylation (OXPHOS) such as alpha-ketoglutarate (alpha-KG) and succinate have been shown to regulate the epigenome (35–37). ISCs undergo epigenetic reprogramming that regulate regeneration and function (Saxena M, Ann Rev Physio 2021). However, whether inflammation regulates OXPHOS and its metabolites in ISCs and if this metabolic adaptation mediates epigenetic reprogramming and function of the ISCs remain unexplored. The concept of epigenetic memory of inflammatory exposure by non-immune cells has been shown for tumor cells (38), and in non-hematopoietic stem cells such as the skin and nasal epithelial stem cells (1–4). It is unknown whether ISCs can retain an epigenetic memory of an inflammatory encounter and whether that modifies their function and sensitivity to future inflammatory encounters.

We investigated whether inflammation induces qualitative metabolic changes in the ISCs and the consequence of such an impact *in vivo*. Utilizing single cell transcriptomics, metabolic analyses, organoid cultures, epigenomics and *in vivo* model systems of GI GVHD we found that inflammation induced metabolic change, reduction in OXPHOS with an increase in succinate leads to epigenetic reprograming of the ISCs, which is retained by the ISCs even in absence of active inflammation. These data thus demonstrate evidence for memory of an inflammatory encounter on by ISCs, provide a molecular mechanism and illustrate a functional consequence on the potential for ISC regeneration.

## Results

### Single cell transcriptomic analysis of Lgr5^+^ISCs following intestinal tract of GVHD

Inflammation-mediated ISC loss is well-documented, however there is little data on functional changes of ISCs that survive the inflammatory insult from GI GVHD. Therefore, to characterize the impact of inflammation on ISCs, we examined the transcriptomes of intestinal crypt epithelial cells using single cell RNA sequencing (scRNA-seq) in the well-established lethally irradiated major histocompatibility complex (MHC)-disparate BALB/c into C57BL/6 (B6) model of GVHD (fig. S1A). The single cells were isolated from the intestines of allogeneic (Allo) (BALB/c→ C57BL/6) and syngeneic (Syn) (C57BL/6→C57BL/6) transplant 7 days after bone marrow transplantation (BMT), and then barcoded with the 10x Chromium technique for scRNA-seq (fig. S1A).

**Figure 1:**
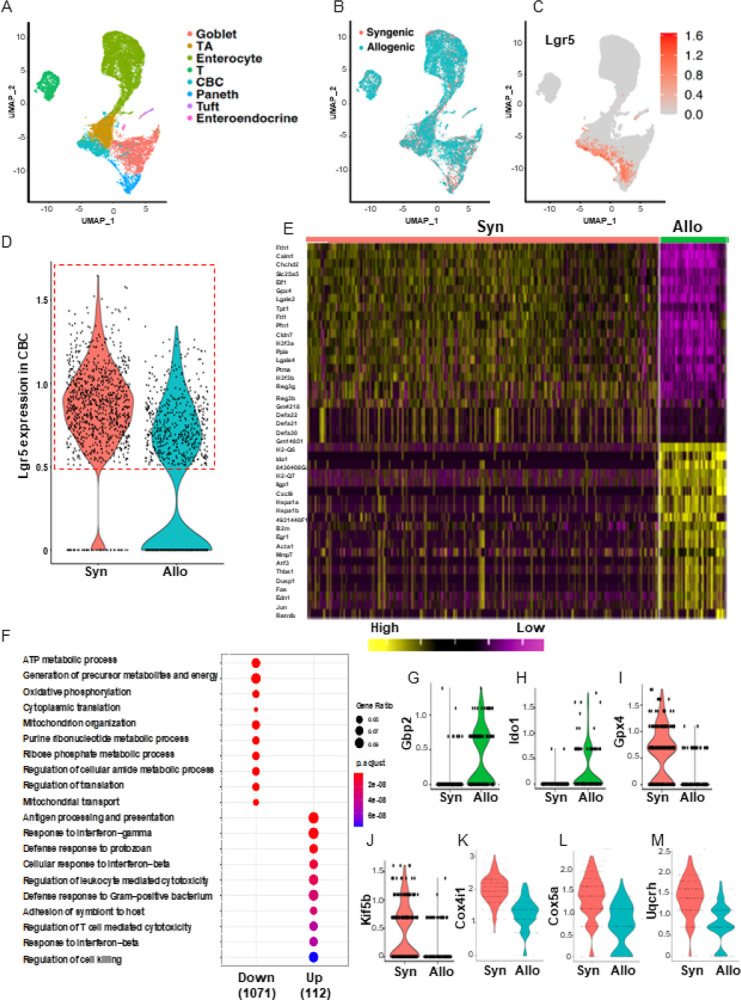
Single cell transcriptomics of Lgr5^+^ISC metabolic on GI GVHD. Single cells were isolated in small intestine crypts from Allo (BALB/c→C57BL/6) or Syn (C57BL/6→C57BL/6) transplant 7 days after BMT, and then barcoded with the 10x Chromium technique for scRNA-seq. n = 2 independent biology samples each group. (A) UMAP plots of distinct clusters with their cell type annotation. Goblet, goblet cells; TA, transient amplifying cells; Enterocyte, absorb enterocytes; T, T lymphocytes; CBC, crypt-base columnar cells; Paneth, Paneth cells; EE, enteroendocrine cells; Tuft, tuft cells; (B) UMAP plots of experimental conditions for sequenced cells; (C) UMAP plots of lgr5 gene expression in clustered cells; (D) Violin plots of lgr5 gene expression in CBC. Clustered Lgr5^+^ISCs are shown within box of red dash line in Syn- and Allo-recipients; (E) Heatmap of top differential genes from Lgr5^+^ISCs cluster between the Syn- and Allo-recipients; (F) Gene ontology enrichment analysis of cellular metabolic process in Lgr5^+^ISCs cluster; (G-M) Representative differentially expressed genes in Lgr5^+^ISCs cluster.

scRNA-seq data was analyzed in R using the Seurat package and integrated using the Seurat canonical correlation workflow (39, 40). All cells in the integrated object were plotted with the Uniform Manifold Approximation and Projection (UMAP) (Fig. 1, A and B). Eight clusters in Allo- and Syn-recipients were identified and annotated based on published gene expression data sets (41, 42), that include the populations of crypt-base columnar cells (CBCs), transit amplifying cells (TA), Paneth cells (Paneth), goblet cells (Goblet), enteroendocrine cells (EE), tuft cells (Tuft), absorb enterocytes (Enterocytes), and T lymphocytes (T) (Fig. 1A and fig. S1B).

To analyze the transcriptomes’ profile of specifically ISCs (fig. S1, B to I), we focused on Lgr5^+^ISCs (Fig. 1C). We used a cut-off of 0.5 and confirmed the Lgr5^+^ISCs population to be within CBC-ISCs by expression of lgr5 (Fig. 1D and fig. S1J). A total of 1128 Lgr5^+^ISCs across the conditions (760 syngeneic condition and 368 allogeneic condition) were identified (Fig. 1D and fig. S1J), and the top differential genes between the Syn- and Allo-recipients were clustered (Fig. 1E). Amongst the highest differentially expressed genes, were the increase in the expression of genes such as *ido1, iigp1, cxcl9, and gbp2* (Fig. 1E, and G to H). By contrast, the most differentially expressed genes that were reduced, were the genes such as *fth1, chchd2, slc25a5, gpx4*, and *kif5b* (Fig. 1, E, I, and J), that are involved in mitochondrial function and cellular metabolic pathways (43–47). 1071 downregulated genes were involved in cellular bioenergetics, including ATP metabolic process, generation of precursor metabolites and energy, oxidative phosphorylation (OXPHOS), cytoplasmic translation, and mitochondrial organization (Fig. 1, F, and K to M). Thus Lgr5^+^ISCs that survived in Allo-GVHD demonstrate differential genes expression in scRNA-seq analysis.

Next, to further confirm these results, we utilized Lgr5-eGFP-IRES-CreERT2 B6 (B6 GFP-Lgr5) animals as BMT recipients and sorted GFP-Lgr5^+^ISCs (Lgr5^+^ISCs) from Allo- or Syn-recipients and validated the differentially expressed genes by qPCR (fig. S1, K to Q). Thus, single cell transcriptomic analysis indicates that Lgr5^+^ISCs that survived in Allo-BMT change their metabolic process, specifically in OXPOS pathway as a response to the inflammation from GVHD.

### Inflammation causes metabolic adaption in ISCs

**Figure 2:**
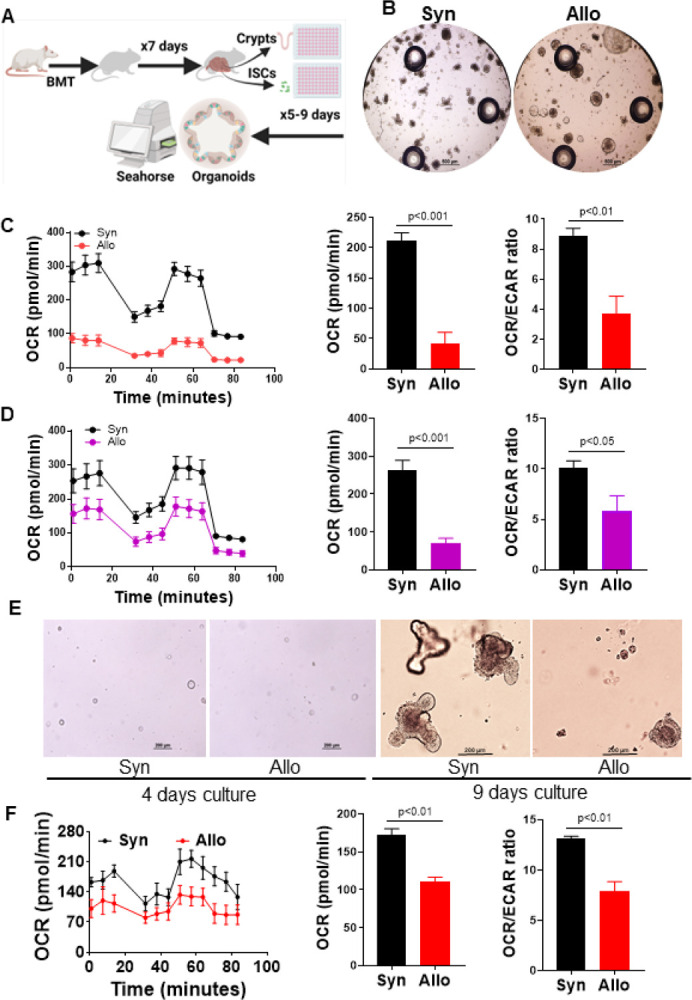
Lgr5^+^ISC-derived organoids from GVHD demonstrate reduced OXPHOS. Small intestinal crypts or their sorted Lgr5^+^ISCs from allogeneic (Allo) (BALB/c→B6 or B6-GFP-Lgr5) or (C3H.SW→B6) and syngeneic (Syn) (B6→B6 or B6-GFP-Lgr5) transplant 7 days after BMT were cultured for organoids formation for five to nine days and then organoids were sequentially exposed to oligomycin, Carbonyl cyanide-4 (trifluoromethoxy) phenylhydrazone (FCCP), and rotenone as well as antimycin A for analyzing oxygen consumption rates (OCRs) via Seahorse platform. (A) Schematic of intestinal organoids culture and Seahorse Assay design. (B-C) Crypts-derived organoids culture, n = 4 Syn (B6→B6), 4 Allo (BALB/c→B6) mice. (B) Representative organoid images per 50 crypts per well cultured for four days, Scale bar, 500 μm. See also Figures S2A; (C) OCR, Basal OCR, and OCR/ECAR ratio on organoids per 50 crypts per well cultured for five days. (D) OCR, Basal OCR, and OCR/ECAR ratio on organoids per 50 crypts per well cultured for five days, n = 4 Syn (B6→B6), 4 Allo (C3H.SW→B6) mice. (E-F) Sorted Lgr5^+^ISCs-derived organoid culture, n = 3 Syn (B6→B6-GFP-Lgr5), 3 Allo (BALB/c→ B6-GFP-Lgr5) independent biology samples. See also Figure 1K. (E) Representative organoid images per 1000 ISCs per well cultured for four to nine days, Scale bar, 200 μm. See also Figures S2D; (F) OCR, Basal OCR, and OCR/ECAR ratio on organoids per 1000 ISCs per well cultured for nine days.

To assess the functional impact of the changes observed in the scRNA analyses, we next utilized the *ex vivo* intestinal organoids culture technique to recapitulate the *in vivo* functional properties of ISCs (48). Because scRNA analyses suggested metabolic alterations in the Lgr5^+^ISCs, to address whether the transcriptomic changes indeed altered the metabolic function of ISCs in context of GI GVHD, we analyzed their bioenergetic profiles by Seahorse XF in organoid cultures (27, 49) (Fig. 2A). Seven days after Syn (C57BL/6→C57BL/6) and Allo (BALB/c→C57BL/6)-BMT, intestine crypts were isolated from allogeneic or syngeneic recipients and cultured in Seahorse 96-well plate with 25–50 crypts per well for five days. Organoid growths were monitored daily and analyzed on day 4 for growth, size and budding. Consistent with previous reports, the numbers of organoids and their buds were significantly decreased in Allo-recipients when compared to that of Syn-recipients (fig. S, A and B), indicating the downregulation of ISCs regeneration capability-mediated by inflammation (15, 50). On day 5 after the initial culture, organoids were sequentially exposed to oligomycin, Carbonyl cyanide-4 (trifluoromethoxy) phenylhydrazone (FCCP) and rotenone, and antimycin A for analyzing oxygen consumption rates (OCRs) via Seahorse platform. Significantly lower levels of OCR and OCR/extracellular acidification rate (ECAR) ratio were observed in organoids derived from Allo-recipients when compared with Syn-recipients (Fig. 2C and fig. S2C).

Next, to confirm whether this observation was generalizable and not limited to a single mouse strain or a model, we generated intestinal organoids from irradiated minor antigen mismatched C3H.SW→B6 model of GVHD and observed once again a significant decrease in OCR and OCR/ECAR ratio in GVHD group when compared with the syngeneic controls (Fig. 2D).

It is formally possible that the reduction in organoids and subsequent differences in OCR might be a result of reduced numbers of Lgr5^+^ISCs in isolated crypts from Allo-recipients. Therefore, to definitively determine that the reduction in organoids and OCR is because of a qualitive change in Lgr5^+^ISCs (and not a reflection of their quantitative loss in the crypts), we directly tested organoid reconstitution and its bioenergetics profiles from sorted Lgr5^+^ISCs in the BALB/c→B6-GFP-Lgr5 model of GVHD. Briefly, seven days after BMT, single cells from small intestine crypts were isolated from Allo- or Syn-recipients and GFP-Lgr5^+^ISCs were sorted by FACS and cultured in Seahorse 96-well plate with 1000 cells per well for 9–10 days and then analyzed by Seahorse. The ISCs-organoid reconstitution was significantly decreased with Lgr5^+^ISCs from Allo-recipients compared with Syn-recipients (Fig. 2E and fig. S2D). Moreover, the level of OCR and OCR/ECAR ratio was also significantly reduced in the organoids reconstituted with Lgr5^+^ISCs from Allo-recipients than that in Syn-recipients (Fig. 2F). Taken together, these findings demonstrate that Lgr5^+^ISCs isolated from allogeneic environment are qualitatively distinct and demonstrate poor regeneration and OXPHOS *ex vivo* even after they have been removed from inflammatory milieu.

### OXPHOS in ISCs regulates severity of GI GVHD

**Figure 3:**
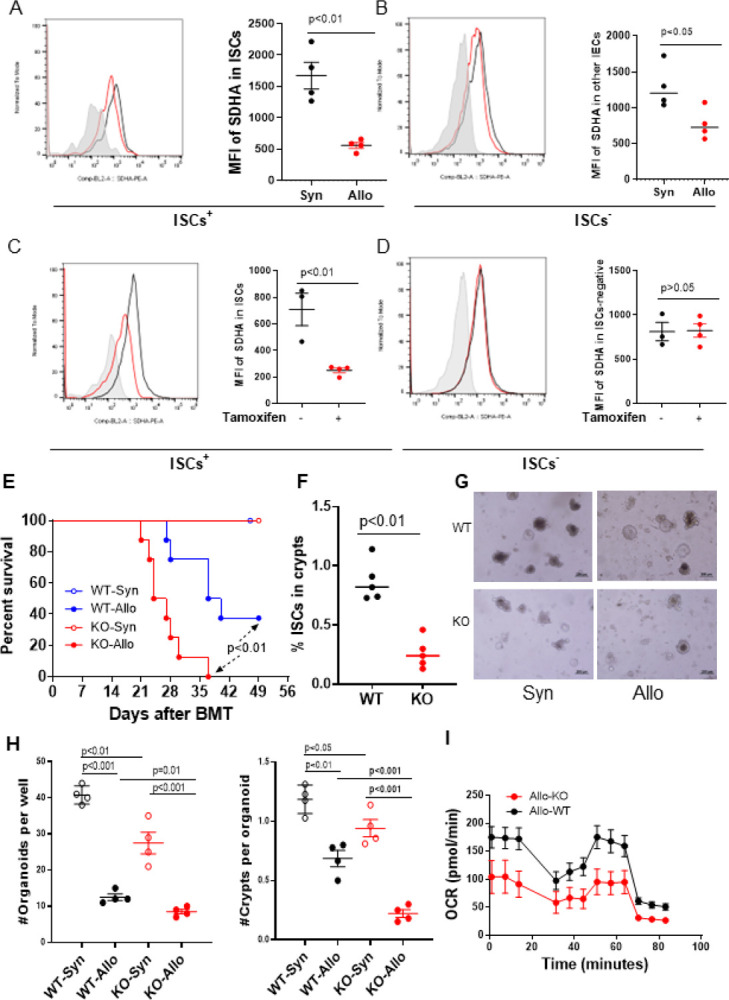
Lgr5^+^ISCs show SDHA deficiency in GI GVHD. (A, B) Single cells were isolated in small intestine crypts from Allo (BALB/c→B6-GFP-Lgr5) or Syn (C57BL/6→B6-GFP-Lgr5) transplant 7 days after BMT, and then SDHA protein analysis in Lgr5^+^ISCs or Lgr5^−^ISCs by flow cytometry, n = 4 mice each group. Representative histograms showing the SHDA expression in Syn (black), Allo (red), control (solid gray). See also Figure S3A. (C, D) Single cells were isolated in small intestine crypts from SdhaΔ/ISC mice treated with tamoxifen or diluent 14 days after initial treatment, and then SDHA protein analysis in Lgr5^+^ISCs or Lgr5^−^ISCs by flow cytometry. n = 3 diluent, 4 tamoxifen treated mice. Representative histograms showing the SHDA expression in Syn (black), Allo (red), control (solid gray). See also Figure S3A. (E-I) SdhaΔ/ISC mice treated with tamoxifen (KO) or diluent (WT) for five days, 14 days after initial treatment, animals received 10-Gy total body irradiation followed by 2.5 × 10^6^ T cells and 5 × 10^6^ BM cells from either syngeneic B6 or allogeneic BALB/c donors. (E) Survival, n = 5 WT-Syn, 8 WT-Allo, 5 KO-Syn, and 8 KO-Allo mice; (F) Lgr5^+^ISCs quantification in small intestine crypts from Allo-WT or Allo-KO seven days after BMT, n = 5 mice each group. See also Figure S1K for ISCs gating strategy. (G-I) Small intestinal crypts isolated from WT or KO recipients 7 days after BMT were cultured for organoids formation for five days and then OCR on organoids were tested by Seahorse, n = 4 mice each group. (G-H) Representative organoid images per 100 crypts per well and quantification of organoids formation, Scale bar, 200 μm; and (I) OCR on organoids per 50 crypts per well cultured for five days.

We next explored whether the reduced OCR in Lgr5^+^ISCs has functional impact *in vivo* GI GVHD severity. Because recent report demonstrated deficiency in SDHA dependent OXPHOS in context of T cell mediated colitis (27) and because Lgr5^+^ISCs show reduced OXPHOS, we first explored the effect of SDHA deficiency on ISCs *in vitro* and *in vivo*. The SDHA protein in Lgr5^+^ISCs was measured from Allo- and Syn-B6 GFP-Lgr5 recipients after BMT by flow cytometry. SDHA expression in Lgr5^+^ ISCs was significantly decreased in Allo-recipients than Syn-recipients, and the magnitude of SDHA loss in Lgr5^+^ ISCs was greater than that in Lgr5^−^ intestinal epithelial cells (IECs) (Fig. 3, A and B) from the Allo-recipients, suggesting that the SDHA protein in ISCs is reduced to a greater extent than in other IECs in the inflammatory milieu of GVHD.

To determine the role of LGR5^+^ISC specific expression of SDHA, we bred Sdha^floxp/floxp^ mice (27) with GFP-Lgr5 mice to generate mice that lack SDHA only in Lgr5^+^ISCs (SdhaΔ/ISC) upon tamoxifen administration (fig. S3A and Fig. 3C). Efficient reduction of SDHA exclusively in the ISCs was confirmed in these SdhaΔ/ISC mice (Fig. 3, C and D). We next utilized tamoxifen-treated ISC-specific knockout (KO) and diluent-treated (WT) SdhaΔ/ISC littermate animals as BMT recipients. Both the KO- and WT-syngeneic recipients survived. By contrast, allogeneic ISC-specific KO-SdhaΔ/ISC-recipients demonstrated significantly greater mortality when compared to the Allo-WT littermate recipients (Fig. 3E). The increase in GVHD mortality was associated with the significant decrease in Lgr5^+^ISCs in the crypts of Allo ISC-specific KO SdhaΔ/ISC-recipients when compared with Allo-WT-recipients (Fig. 3F). However, no significant differences were noted in donor T cell expansion, Tregs or cytokine production (fig. S3, B to E).

These data demonstrated that the alteration in SDHA dependent OXPHOS in Lgr5^+^ISCs regulates GVHD severity *in vivo*.

Next, we compared the organoid formation by the harvested intestinal crypts derived from the ISC-specific KO SdhaΔ/ISC recipients and WT recipients (Fig. 3, G to H). Interestingly, the genetic deletion of SDHA in ISCs significantly reduced the numbers of organoids and buds in both ISC-specific KO SdhaΔ/ISC Allo- and Syn-recipients when compared with WT-recipients (Fig. 3, G to H). But the reduction was much greater in the KOs than the WT Allo-recipients. Seahorse assay further showed a significant decrease of OCRs in Allo-KO-recipients than Allo-WT-recipients (Fig. 3I), directly demonstrating that Lgr5^+^ISC intrinsic alteration in SDHA dependent OXPHOS enhanced intestinal tract sensitivity to inflammation induced damage during GVHD.

### Succinate regulates ISC function and DNA methylation

**Figure 4:**
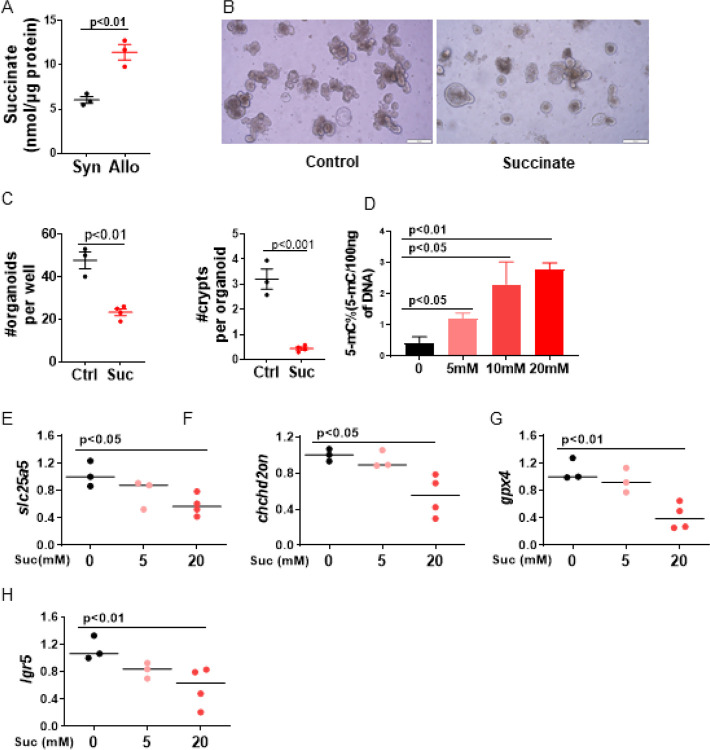
Succinate accumulation regulates ISC function and DNA methylation. (A) Intestine organoids cultured for five days from Allo (BALB/c→B6) and Syn (B6→B6) in Figures 2 were harvested and quantified for succinate level. n = 4 mice, each group. (B-H) Intestine organoids cultured for three days from naïve GFP-Lgr5 mice and then treated with dimethyl succinate or diluent control for seventy-two hours. (B-C) Representative organoid images and quantification per 100 crypts per well. Scale bar, 200 μm, Ctrl, diluent control; Suc, 20mM of dimethyl succinate, n = 3 independent biology samples each group; (D) 5-mC quantification of organoids, n = 4 independent biology samples each group; (E-H) Gene expression in organoids, n = 3 independent biology samples each group.

OXPHOS is a critical process that generates ATP and is indispensable for ISC fate decisions (17, 19, 23, 51–53). To understand the relationship of SDHA dependent metabolism and Lgr5^+^ISCs’ regeneration capability in context of GVHD, we next quantified succinate levels, a metabolic byproduct of tricarboxylic acid (TCA) cycle that accumulates in context of SDHA deficiency (27). We observed significantly increased levels of succinate in organoids from Allo-recipients than from Syn-recipients (Fig. 4A), consistent with the reduction in SDHA and OCR (27).

Succinate induces DNA methylation, therefore, we next explored whether succinate accumulation directly regulated Lgr5^+^ISCs regeneration and its DNA methylation (54, 55). To this end, we treated organoids with cell-permeable dimethyl succinate or diluent control to directly evaluate the impact of succinate on organoid formation and epigenome. Seventy-two hours after the succinate treatment, we observed organoids formation from sorted Lgr5^+^ISCs was inhibited (Fig. 4B–4C). The amount of global DNA methylation (5-mC) in organoids measured by assessment of 5-mC levels was significantly increased after treatment with succinate when compared with control organoids (Fig. 4D). To investigate whether succinate treatment directly regulated gene expression as observed from the transcriptomic analysis from above, we analyzed the expression of *slc25a5, chchd2, gpx4*, and *Lgr5* with qPCR (Fig. 4, E to H). Their expression was significantly reduced in succinate treated organoids when compared with control treated organoids. Thus, the genes that were downregulated from the Lgr5^+^ISCs transcriptomes analysis in context of GVHD were also downregulated by succinate treatment suggesting that alteration of SDHA dependent metabolism during GVHD regulates ISC transcriptome.

### Lgr5^+^ISC epigenome is reprogramed in context of GI GVHD

**Figure 5:**
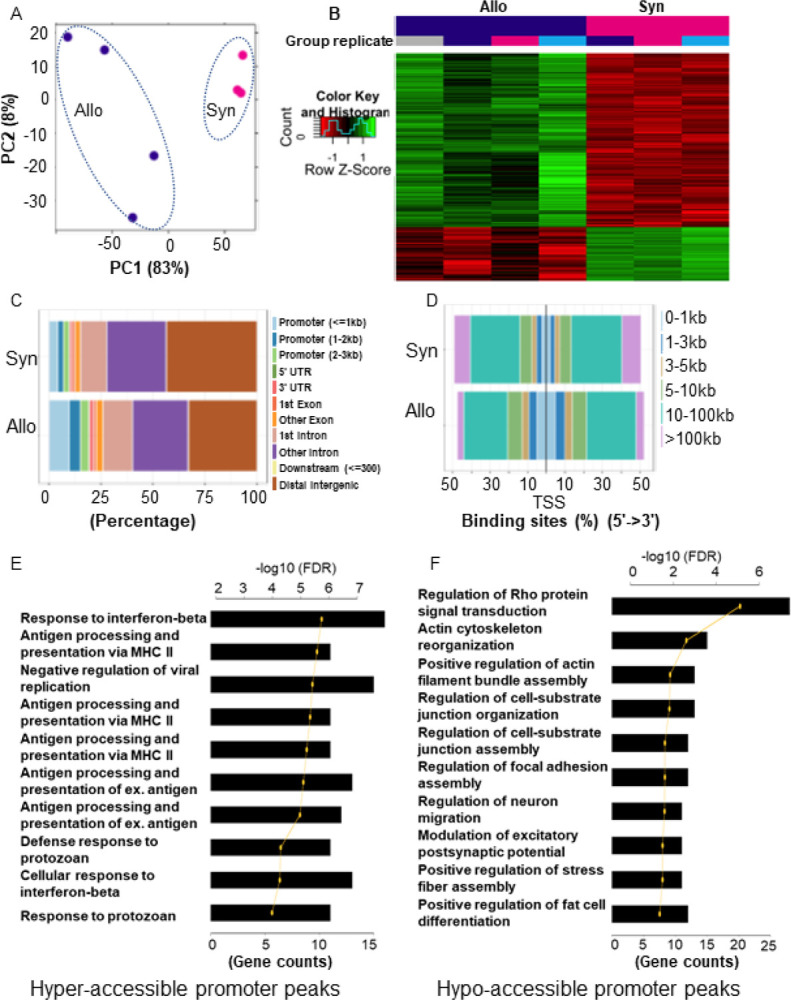
ISCs epigenome is altered in context of GI GVHD. Lgr5^+^ISCs were sorted as Figure S1K from Allo (BALB/c→B6-GFP-Lgr5) and Syn (B6→B6-GFP-Lgr5) transplant 7 days after BMT and performed for ATAC-seq, n = 3 Syn (B6→B6-GFP-Lgr5), 4 Allo (BALB/c→B6-GFP-Lgr5) independent biology samples. ATAC-seq data were analyzed for the peaks of chromatin accessibility. (A) Principal component analysis (PCA) plot of peaks, (B) Heatmap of differential peaks, (C) Annotation of differential peaks based on their positions in genome, (D) Distance of peaks to closest transcription start sites (TSS), (E) GO enrichment analysis of hyper-accessible promoter peaks, (F) GO enrichment analysis of hypo-accessible promoter peaks in Allo-recipients compared to Syn-recipients.

Because succinate, the byproduct of the metabolic deficit in ISCs after Allo-BMT, modified DNA methylation and regulated organoid growth, we next determined whether ISC epigenome was reprogramed after Allo-GVHD. To this end, we performed ATAC-seq in sorted Lgr5^+^ISCs harvested from the BALB/c→B6-GFP-Lgr5 model of GVHD. Briefly, fifty thousand Lgr5^+^ISCs were sorted by FACS from Allo- and Syn-recipients seven days following BMT as above. To generate the ATAC-Seq library, we used the Omni-ATAC-Seq protocol (56). The libraries were pooled, quantitated, and sequenced. ATAC-seq data were analyzed using the Diffbind package in R, and showed a change in landscape, including increased chromatin accessibility in 6252 peaks of and a reduction in chromatin accessibility in 3167 peaks in Allo-recipients, compared with Syn-recipients (Fig. 5, A and B and fig. S4A). To further explore the position of genome chromatin accessibility, we annotated the peaks using the ChipSeeker R package and split the reads based on differential accessibility in all conditions. We observed that there was a difference in distance of peaks to closest transcription start sites (TSS) and different binding sites (Fig. 5, C and D) between Allo- and Syn-recipients.

To understand which signal pathway was involved in the chromatin sites that were differential accessible, we performed Gene Ontology (GO) enrichment analysis using Protein ANalysis THrough Evolutionary Relationships (PANTHER). Mapping the hyper-accessible regions of the corresponding genes (3kb neighborhood of TSS) resulted in 1493 gene targets, most of which are known to be involved in immune responses and antigen processing and presentation (Fig. 5E). In contrast, the corresponding genes to the hypo-accessible regions are mostly related to the Rho GTPase pathway and actin cytoskeleton pathway, which participate in a wide variety of cell processes including proliferation and adhesion (Fig. 5F). Importantly, the epigenetic changes from the ATAC-seq were consistent with expression of genes observed in transcriptomic analysis such as *ido1, gbp2, gpx4*, and *kif5b* (fig. S4, B to E) after Allo-BMT.

### Epigenetic reprograming impacts ISC memory

**Figure 6:**
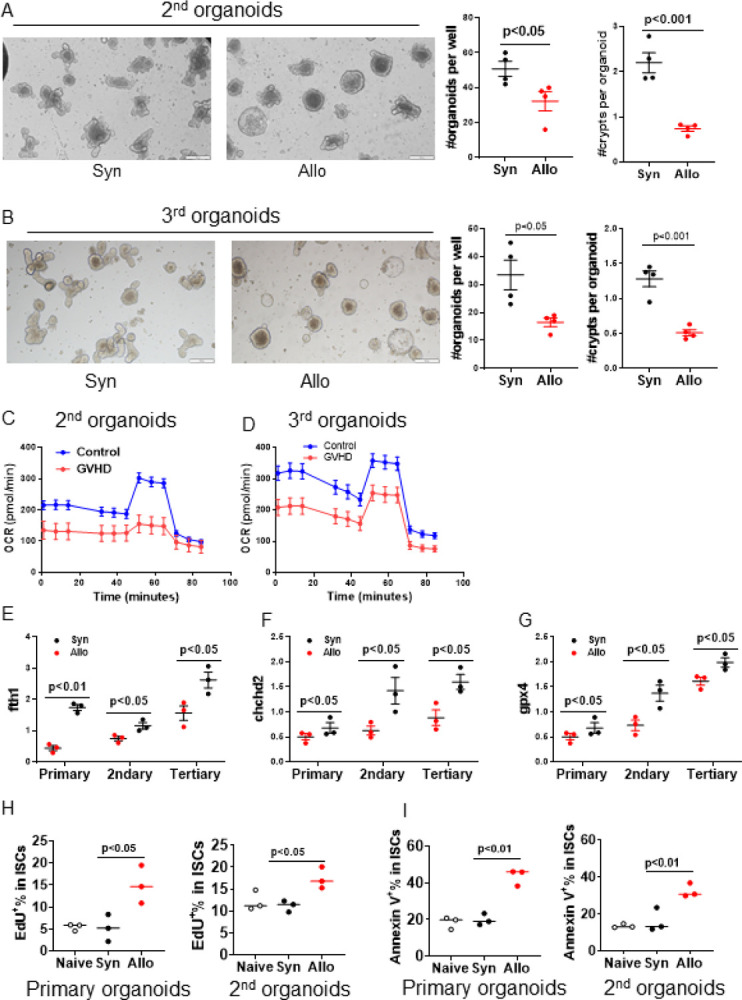
Effects of epigenetic reprograming is persistent through serial organoid cultures. (A-G) Primary intestine organoids as in Figures 2 from Allo (BALB/c→ B6) and Syn (B6→B6) were serial passaged and their OCR were tested via Seahorse. Each generation of organoids were harvested for testing genes expression by qPCR. (A) Representative the secondary organoid images per 100 crypts per well cultured for four days and quantification, Scale bar, 200 μm, n = 4 mice each group; (B) Representative the tertiary organoid images per 100 crypts per well cultured for four days and quantification, Scale bar, 200 μm, n = 4 mice each group; OCR assay from the secondary organoids (C) and the tertiary organoids (D) per 50 crypts per well cultured for five days, n = 3 each mice group; (E-G) Gene expression in organoids, n = 3 mice each group. (H-I) Primary intestine organoids as in Figures 2 from Allo (BALB/c→B6-GFP-Lgr5) and Syn (B6→B6-GFP-Lgr5) or from naïve B6-GFP-Lgr5 mice were serial passaged and were labeled with EdU for quantification EdU^+^Lgr5^+^ISCs as in methods, n = 3 each mice group. (H) Quantification of EdU^+^Lgr5^+^ISCs; (I) Quantification of Annexin V^+^Lgr5^+^ISCs.

We next analyzed whether the Lgr5^+^ISCs retain the memory of the functional deficits induced by epigenome reprograming. We hypothesized that the reduction in organoid formation and OCR will be retained in serial organoid passages. To determine the functional deficit, Lgr5^+^ISC crypts harvested from Syn- and Allo-recipients were harvested and placed in organoid cultures as above. Five days later, these primary organoids cultures were split and passaged for the next generation to determine whether the deficits in Allo-organoids persisted in subsequent generations of organoids following secondary and also tertiary passage. We monitored organoid growth daily and evaluated their organoids formation and bioenergetics from Allo- and Syn-recipients. The numbers of organoids and their buds were significantly decreased in Allo-recipients compared to Syn-recipients in primary organoids and also in the secondary and tertiary passaged organoids (fig. S2, A and B and Fig. 6, A and B). Seahorse assay showed the reduction of OCRs observed in primary organoids from Allo-recipients was sustained at a significantly lower level in the secondary and tertiary organoids than the Syn-recipients (Fig. 6, C and D). Furthermore, the lower expression level of *fth1, chchd2*, and *gpx4*-expression levels in ISCs after GVHD were also reduced in primary, secondary, and tertiary organoids in Allo-recipients compared to Syn-recipients in serial organoids cultures (Fig. 6, E to G). Thus, ISCs from Allo-animals demonstrated persistent deficits in their organoid regeneration, OCR and gene expression indicating that epigenetic rewiring of the ISCs, induced by their encounter with inflammation from GVHD *in vivo*, is remembered by the ISCs for multiple generations despite the absence of active inflammation in the *ex vivo* organoid milieu.

We next analyzed whether the persistent cellular impact induced from the inflammatory encounter by the Lgr5^+^ISCs is linked to epigenetic reprograming. Because 5-ethynyl-2’-deoxyuridine (EdU)-labeling of Lgr5^+^ISCs in crypts were increased *in vivo* in the GVHD recipients compared to no-GVHD recipients (57), and because organoids showed reduction in growth *ex vivo*, we next analyzed whether the high fold changes (FC) of chromatin accessibility peaks were noted in genes that regulate cell cycle and cell biosynthetic process (fig. S5A). We observed upregulation of gene signatures for G2-M check point and inflammation response pathways in Lgr5^+^ISCs by scRNA-seq (Fig. S5, B and C). Therefore, to determine whether the epigenome changes had functional impact on ISCs that encountered inflammation and survived *in vivo*, we analyzed Lgr5^+^ISCs proliferation in serial organoids culture from Allo- and Syn-recipients of GFP-Lgr5 mice. We pulsed EdU into organoids culture for 1 hour and then analyzed EdU^+^Lgr5^+^ISCs by FACS (see Methods and fig. S6A). We observed significantly higher percentage of EdU^+^Lgr5^+^ISCs in Allo-recipients in primary and secondary organoids compared with Syn-recipients (Fig. 6H). Furthermore, higher levels of Annexin V^+^Lgr5^+^ISCs was observed in Allo-recipients in both primary and secondary organoids compared with Syn-recipients (Fig. 6I and fig. S6B). Thus inflammation-mediated epigenetic reprograming altered Lgr5^+^ISCs propensity for proliferation and apoptosis that persisted in serial organoid cultures.

Next, to directly link metabolic deficit to epigenome induced cellular function changes we utilized malonate, a SDHA inhibitor, in organoids culture as described in the methods, and assessed for changes in organoids formation and Lgr5^+^ISCs proliferation. The organoids formation was reduced (fig. S7A) and the percentage of EdU^+^Lgr5^+^ISCs and Annexin V^+^Lgr5^+^ISCs were increased in malonate-treated organoids compared to control (fig. S6, A and C, and S7, B and C). To rule out impact of off-target effect of malonate, and to further confirm that the metabolic deficit in SDHA regulated Lgr5^+^ISCs proliferation *in vivo*, we once again utilized ISC-specific KO SdhaΔ/ISC mice as above. Fifteen days after treatment of tamoxifen, we pulsed EdU for 2 hours and then harvested small intestine to analyze EdU^+^Lgr5^+^ISCs by FACS (fig. S6, A to C). We observed that the percentage of EdU^+^Lgr5^+^ISCs were substantially higher in the Sdha-deleted mice compared with Sdha-intact SdhaΔ/ISC mice that were treated with diluent (fig. S7D). Furthermore, the percentage of Ki67^+^Lgr5^+^ISCs and Annexin V^+^Lgr5^+^ISCs were significantly increased in Sdha-deleted mice than Sdha-intact mice (Fig. S7, E and F). Taken together, these results demonstrate inflammation mediated-metabolic alteration in SDHA, and the subsequent-succinate accumulation induced epigenetic rewiring result in Lgr5^+^ISCs dysfunction that are retained and regulate the severity of GI GVHD.

## Discussion

Adult Lgr5^+^ISCs reside at the bottom of the crypt and are critical for the regeneration and repair of intestinal epithelium at homeostasis and following injury (6, 58). Lgr5^+^ISCs display context-dependent functionality and adapt to different requirements over their lifetime, as dictated by their microenvironment cues (7, 22). Cellular response to inflammation-induced stress in ISCs and its impact on their long-term ability is not well understood. Because emerging data demonstrate that adult stem cells harbor inflammatory memory, we examined whether Lgr5^+^ISCs might also possess an inflammatory memory and if so, its mechanisms and their cellular functional impact (1–4). Utilizing well-characterized *in vivo* models of Allo-BMT wherein ISCs are known to be bona fide targets of GI GVHD inflammation, we found that inflammation induced OXPHOS deficiency in Lgr5^+^ ISCs leading to accumulation of succinate, that reprogrammed the epigenome and regulated their ability to regenerate in multiple passages of organoid development.

GI GVHD is known to be caused by the depletion of ISCs caused by alloreactive T cell induced inflammation in the context of intestinal microbiome and innate immune activation (8, 10). Several recent studies have explored the cause and impact of loss of ISCs *in vivo*, and *ex vivo* in organoid cultures, by their inability to grow, bud and differentiate (12–15). However, whether GI GVHD inflammation induces any qualitative changes in the ISCs besides the quantitative change caused by their apoptosis remained unclear. Our data demonstrate that GI GVHD induced inflammation causes qualitative changes in the ISCs. Specifically, single cell transcriptomics show significant changes in the transcriptomics of the harvested ISCs the allogeneic recipients. We found significant gene downregulation in the pathways of ATP metabolic process, generation of precursor metabolites and energy, and OXPHOS. The qualitative differences in the Lgr5^+^ ISCs from the GVHD and control recipients ISCs were observed in the organoid reconstitution and crypts organoids formation from the sorted post-BMT Lgr5^+^ISCs in the absence of ongoing ex vivo inflammation. Furthermore, consistent with reduction in pathways related to OXPHOS and ATP production from the single cell transcriptomic analysis, the Lgr5 + ISCs from GVHD also demonstrated a reduction in their OCRs indicating a functional metabolic defect.

Metabolic alterations induced from and by inflammation in immune cells and tumors is increasingly appreciated (59, 60). Whilst adult stem cells may have a metabolic signature that is distinct from more differentiated cells (61, 62), our demonstrate a similar metabolic defect in SDHA dependent OXPHOS metabolism in Lgr5^+^ISCs as was reported in IECs (27), albeit to an even greater degree of reduction in SDHA in the ISCs. Interestingly, Lgr5^+^ISCs show a higher OXPHOS activity compared to other differentiated epithelial cells (51, 63, 64) thus, ISCs may be more dependent on SDHA. However, whether greater levels of SDHA in ISCs than the IECs makes them more sensitive to alloreactive T cell mediated inflammation will need to be analyzed in future studies. Nonetheless our data unequivocally demonstrate that SDHA in Lgr5^+^ISCs modulates organoid formation and, also regulates GI GVHD severity *in vivo*, thus demonstrating it to be a critical inflammation induced metabolic checkpoint in ISCs and IECs. ISCs include several subtypes which may play a context dependent role (42, 65–68). It is important to note that our study focused on, and therefore is limited only to the Lgr5^+^ISCs, which are the actively cycling-type stem cells and are sensitive to irradiation and inflammation (65, 69, 70). Furthermore, progenitor intestinal cells and mature cells subtypes such as Paneth cells demonstrate metabolic heterogeneity (6, 71, 72). Therefore, the impact of inflammation on SDHA induced metabolic checkpoint on various intestinal epithelial cell subsets may be distinct and will also need to be determined in future studies.

Our data demonstrates that reduction in SDHA led to accumulation of succinate as a byproduct of inflammation-induced metabolic adaptation in Lgr5^+^ISCs. Succinate is a known oncometabolite that affects DNA methylation in tumors, affects monocyte/macrophage responses and promotes post-translational modification of histones and other proteins (54, 73, 74). Our data now link succinate to ISC epigenetic remodeling and cellular functions. Succinate accumulation impaired organoids formation and increased DNA methylation in organoids. ATAC-seq of ISCs showed changes driven by the alteration in DNA methylation after exposure to inflammation. The epigenomic changes are demonstrated by changes in the chromatin accessibility peak changes in their position and binding sizes of genes that could regulate ISC growth and differentiation. Integration with transcriptomics showed that the changes in epigenome most significantly regulated genes that affect cell cycling. These changes in gene expression were confirmed in SDHA deleted Lgr5^+^ISCs, thus linking succinate with epigenetic regulation of cell cycling genes. The differential impact of succinate accumulation on DNA methylation and the effects on post-translational modification of proteins in ISCs or any other cell types remain to be addressed. Nonetheless, utilizing serial organoids culture, we demonstrate that the changes in the expression of these genes, and the functional impact on cell cycling persist, suggesting that the rewiring the epigenome of ISCs is retained following their exposure to inflammation *in vivo*.

To our knowledge, this is a first demonstration of inflammation induced metabolic change that remodels the epigenome and leaves its memory in ISCs. Whether similar or distinct reprograming occurs in other tissues and other types of inflammation will need to be analyzed. Importantly, metabolic adaptations in OXPHOS play a role in generation of immune memory (75–78). When considered in light of our study it suggests that OXPHOS is one common mechanism of epigenetic reprogramming that regulates memory in both immune and non-immune cells such as ISCs. However, whether the impact on cellular memory by OXPHOS alterations is specifically only from an SDHA dependent process, or whether the mechanisms are variable, or distinct depending on the type of stimuli will need to be addressed in future studies. Regardless, because our data suggests the epigenetic rewiring persists even after inflammation subsides, the residual effects on ISCs may affect their fitness and subsequent tolerance of future stressors thus having critical implications for intestinal tissue resilience and repair.

Cellular adaptation is a fundamental cellular property that maximizes fitness to environmental pressures. ISCs are exposed to a number of environmental cues that regulate their fitness, function and fate at homeostasis. Therefore, whether the environment of ISCs contributes to its epigenetic imprinting induced by inflammatory metabolic changes and if that determines the maladaptive or a protective nature of the inflammatory memory remains to be investigated. A recent seminal study demonstrated that exposure to an infection during pregnancy triggered inflammatory memory within in fetal intestinal epithelial cells and protected the offspring from later infection demonstrating protective intergenerational memory in the intestinal epithelial cells (3). Our data now extend the notion of inflammatory memory to ISCs and suggest that a memory may be detrimental or maladaptive because of the persistent defect in the ability of organoid formations by these ISCs in serial organoid cultures. Our data suggest that the ISCs retain the scars of inflammation, and these impair their ability to regenerate and repair. Thus, following an inflammatory encounter, the GI tract may become more sensitive to the next stressor or even to homeostatic environmental cues. Our data therefore suggest that next generation of therapies targeting metabolic adaptation of epigenetic rewiring could allow for better tissue tolerance and regeneration in the context of immune mediated damage.

## Data Availability

All data are available in the main text and the supplementary materials.
